# Impact of the Warburg effect on nucleotide homeostasis in human retinal endothelial cells and its relevance to proliferative diabetic retinopathy

**DOI:** 10.3389/fphar.2025.1660067

**Published:** 2025-11-03

**Authors:** Andrew Gregory, Ahmed M. Awad, Shaimaa Eltanani, Thangal Yumnamcha, Jenna Hart, Rao Me, Xihui Lin, Mohamed Shawky, Ahmed S. Ibrahim

**Affiliations:** 1 Department of Ophthalmology, Visual, and Anatomical Sciences, Wayne State University, Detroit, MI, United States; 2 Department of Pharmacology and Toxicology, Faculty of Pharmacy, Mansoura University, Mansoura, Egypt; 3 Department of Pharmacology and Toxicology, Faculty of Pharmacy, Mansoura National University, Mansoura, Egypt; 4 Department of Pharmacology, School of Medicine, Wayne State University, Detroit, MI, United States; 5 Molecular Therapeutics Research Program, Karmanos Cancer Institute, School of Medicine, Wayne State University, Detroit, MI, United States

**Keywords:** proliferative diabetic retinopathy (PDR), the Warburg effect, nucleotide metabolism, human retinal endothelial cells (HRECs), high glucose (HG), hypoxia (Hyp), biomarkers, nucleoside monophosphates (NMPs)

## Abstract

**Purpose:**

While great progress has been made in screening methods and therapies for proliferative diabetic retinopathy (PDR), it is still a major cause of blindness. Rapidly dividing cells reprogram their metabolism toward hyperglycolysis (the Warburg effect), a process recently implicated in angiogenesis. In this study, we sought to investigate nucleotide metabolism in human retinal endothelial cells (HRECs) under high glucose (HG) and hypoxia (Hyp), both key risk factors in PDR and known to induce the Warburg effect, and to validate these findings in patients with PDR.

**Methods:**

HRECs were cultured under normal conditions and then exposed to HG, Hyp, or both. Metabolomic profiling was performed using liquid chromatography-mass spectrometry (LC-MS/MS) to quantify nucleotide-related metabolites. In parallel, proteomic analysis was conducted to assess proteins involved in nucleotide metabolism. To validate the *in vitro* findings, vitreous samples from patients with PDR and non-PDR controls were analyzed. Receiver operating characteristic (ROC) analysis was then applied to evaluate the diagnostic potential of nucleotide metabolites in PDR.

**Results:**

HG and Hyp in HRECs caused selective disruptions in nucleotide metabolism, marked by significant accumulation of D-ribose-5-phosphate, a glycolytic precursor for both purines and pyrimidines, as well as nucleoside mono- and diphosphates (NMPs, NDPs), particularly adenosine mono- and diphosphates (AMP and ADP), without global changes in total nucleotide pools. This accumulation was also observed in vitreous samples from patients with PDR. ROC analysis identified AMP+ADP levels >0.0062 µM as a potential diagnostic biomarker for PDR with 87.5% specificity. Proteomic profiling revealed dysregulation of key enzymes regulating nucleotide homeostasis, including reduced expression of mitochondrial nucleoside diphosphate kinase (NME4), increased levels of cytosolic adenylate kinase (AK1), and upregulation of multiple enzymes involved in *de novo* and salvage nucleotide biosynthesis. Notably, expression of ribonucleotide reductase catalytic subunit M (RRM2), which converts NDPs to deoxynucleotides (dNDPs) for DNA synthesis, was also elevated.

**Conclusion:**

Exposure to HG and Hyp, key risk factors for PDR, disrupts nucleotide homeostasis in HRECs, with enhanced glycolytic flux fueling nucleotide precursor production and altered kinase expression favoring the accumulation of nucleoside mono- and diphosphates over triphosphates. The corresponding increase in AMP and ADP in PDR vitreous highlights their potential as biomarkers and underscores the central role of nucleotide metabolism in PDR pathogenesis.

## Introduction

The incidence of diabetes is rising globally, and by 2030, over 50 million Americans are projected to be affected (Saeedi et al., 2019). Diabetic retinopathy (DR) develops in approximately 27% of individuals with diabetes and can progress to proliferative DR (PDR), a leading cause of blindness (Lundeen et al., 2023). In PDR, the retina undergoes retinal neovascularization (RNV), in which disorganized new blood vessels form, ultimately causing vision loss through bleeding and retinal detachments (Jampol et al., 2020). Current therapies for PDR, including laser photocoagulation and intravitreal anti-vascular endothelial growth factor (VEGF) injections, have limitations. Laser treatment can lead to temporary or permanent vision changes, such as blurred vision or reduced night vision (Pande and Tidake, 2022), while anti-VEGF therapy may increase the risk of thromboembolic events and retinal detachment (Schlenker et al., 2015; Zehden et al., 2022). These limitations underscore the need to better understand the mechanisms driving RNV to identify novel pathways and develop more effective or complementary treatments.

In the field of PDR, it is not entirely clear why RNV develops only after many years of diabetes. One hypothesis proposes that PDR involves a multifaceted set of processes, in which hyperglycemia alone is not the sole driver; tissue hypoxia (Hyp), arising from retinal capillary nonperfusion, emerges as another significant contributor to RNV in PDR (Catrina and Zheng, 2021; Arden and Sivaprasad, 2011). Several lines of clinical evidence support this hypothesis. Ultra-widefield fluorescein angiography (UWF-FA) reveals progressive areas of capillary nonperfusion (quantified as the nonperfusion index), which strongly correlate with disease severity and the risk of neovascularization in PDR (Silva et al., 2022). Similarly, optical coherence tomography angiography (OCTA) studies show reduced capillary perfusion density and enlargement of the foveal avascular zone (FAZ) in DR, with more pronounced changes in eyes that progress to PDR (Yang et al., 2025). Retinal oximetry also demonstrates altered oxygen saturation and impaired oxygen delivery in DR, reflecting Hyp-related metabolic imbalance; specifically, individuals without diabetes show higher retinal oxygen saturation than those with mild DR, who in turn exhibit higher saturation than patients with moderate to severe DR (Hommer et al., 2022). At the molecular level, Hyp in PDR results from a combination of structural and cellular alterations, including pericyte loss, glial activation, thickening of the basement membrane, and capillary dropout (Arden and Sivaprasad, 2011; Feit-Leichman et al., 2005; Linsenmeier et al., 1998). These changes collectively reduce oxygen delivery to retinal tissues to stabilize hypoxia-inducible factors (HIF1/2α), key transcription factors induced in response to low O_2_ levels to regulate glucose homeostasis and glycolysis (Semenza, 2007; Hu et al., 2003). In support, HIFs have been detected in neovascular tissue of PDR eyes even with low VEGF levels, highlighting the contribution of Hyp-driven VEGF-independent pathways (Qin et al., 2022).

Unlike other diabetic complications, no rodent model to date fully replicates human PDR. The mouse oxygen-induced retinopathy (OIR) model is commonly used to study Hyp-driven RNV, but its lack of hyperglycemia limits its relevance to PDR (Scott and Fruttiger, 2010). The Akimba mouse model, generated by crossing the Ins2Akita diabetes model with the Kimba VEGF-overexpressing mouse, develops RNV in the context of hyperglycemia (Rakoczy et al., 2010). However, this model is primarily VEGF-driven and fails to capture Hyp-dependent non-VEGF pathways in PDR angiogenesis, such as those mediated by erythropoietin and angiopoietins (Scholz et al., 1990; Simon et al., 2008). Therefore, models integrating both hyperglycemia and Hyp are needed to better recapitulate human PDR and uncover new therapeutic targets. Our high glucose (HG) + Hyp human retinal endothelial cells (HRECs) model addresses this gap by incorporating two central pathophysiological drivers of PDR, hyperglycemia and Hyp, within a controlled experimental setting. While this *in vitro* system cannot fully reproduce the complexity of human PDR, including the roles of other retinal cell types and systemic influences, it provides a tractable platform to dissect molecular mechanisms, identify candidate metabolic pathways, and generate hypotheses for future *in vivo* validation (Gregory et al., 2023).

Under physiological conditions, endothelial cells rely on the coordinated interplay of multiple metabolic programs. Glycolysis provides rapid adenosine triphosphate (ATP) production, while mitochondrial oxidative phosphorylation, supported by the tricarboxylic acid (TCA) cycle, glutaminolysis, reductive carboxylation of glutamine, and fatty acid oxidation, supplies sustained energy essential for maintaining blood–retinal barrier integrity (Oska et al., 2025; Oska et al., 2023; Eltanani et al., 2022). In contrast, under conditions such as Hyp and hyperglycemia that drive pathological angiogenesis, HIFs promote a metabolic shift favoring hyperglycolysis over oxidative phosphorylation, a defining feature of the Warburg effect (Warburg, 1956). HIFs support this adaptation by upregulating glycolytic enzymes, including aldolase A, phosphoglycerate kinase (PGK)1, and pyruvate kinase (Semenza et al., 1994), as well as glucose transporter (GLUT)1 to enhance glucose uptake (Ouiddir et al., 1999) and lactate dehydrogenase (LDH) to convert pyruvate to lactate (Semenza et al., 1994). Concurrently, HIFs suppress TCA cycle activity through pyruvate dehydrogenase kinase (PDK)1 induction (Kim et al., 2006) and reduce fatty acid β-oxidation by downregulating carnitine palmitoyltransferase (CPT)1 (Du et al., 2017). While glutaminolysis normally supplies α-ketoglutarate to the TCA cycle, this oxidative contribution is curtailed under Hyp, with glutamine redirected toward reductive carboxylation to support lipid synthesis and redox balance (Fendt et al., 2013). Hyperglycemia further amplifies glycolytic flux by providing abundant glucose, leading to increased glycolytic flux and lactate accumulation. Collectively, these changes establish hyperglycolysis as one of the dominant adaptations under dual Hyp and HG stress (Gregory et al., 2023). This metabolic shift is consistent with the Warburg effect, a well-established phenomenon in cancer, where cells generate ATP rapidly, though less efficiently than through oxidative phosphorylation, while simultaneously producing intermediates that fuel anabolic pathways required for cell proliferation (Liberti et al., 2016).

Among these pathways, nucleotide metabolism was identified as one of the top significantly altered pathways in HRECs exposed to combined HG and Hyp, based on pathway enrichment analysis using the Small Molecule Pathway Database (Supplementary Figure S1). Nucleotide homeostasis is particularly important in this context, as nucleotides not only provide the building blocks for DNA and RNA but also act as energy carriers and signaling molecules, directly supporting endothelial proliferation and angiogenesis. Structurally, each nucleotide consists of three main components: a nitrogenous base (adenine, guanine, cytosine, thymine, or uracil), a five-carbon sugar (ribose in RNA or deoxyribose in DNA), and one or more phosphate groups. Nucleotides are further subdivided according to the nitrogenous base into purines and pyrimidines. The purines, made up of 2 heterocyclic aromatic rings, include adenine and guanine, while the pyrimidines, made up of 1 heterocyclic aromatic ring, include cytosine, uracil, and thymine. Cytosine, adenine, and guanine are shared between DNA and RNA molecules, while thymine and uracil are nucleotide bases specific to DNA and RNA, respectively. In the context of blood vessel growth in tumors (Zulato et al., 2012) and diabetic nephropathy (Wang et al., 2022), the Warburg effect has been recognized as a significant driving factor. However, its impact on the nucleotide metabolism of retinal endothelial cells under diabetic and hypoxic conditions remains poorly understood.

The present study aimed to characterize the effects of combined HG and Hyp on nucleotide profiles in HRECs. We further identified dysregulated pathways based on significantly altered nucleotides and validated these findings in the vitreous humor of patients with PDR. Characterizing these alterations may facilitate the development of targeted therapies and the discovery of clinically relevant biomarkers for PDR.

## Materials and methods

### *In vitro* cell culture

Human retinal endothelial cells (HRECs) were obtained from Cell Systems (Kirkland, WA, USA), which provides cells isolated from healthy donors, and cultured in Microvascular Endothelial Cell Growth Medium-2 Bullet Kit (Lonza, Walkersville, MD, USA; Catalog No. CC-3202 EGM-2 MV), which contains 5 mM D-glucose, supplemented with 5% fetal bovine serum (FBS) and growth factors as previously described (Gregory et al., 2023; Eltanani et al., 2022). Cells at passages 3–9 were seeded in 100 mm Petri dishes and cultured until approximately 90% confluency was reached. For experimental treatments, the medium was replaced with fresh medium containing 5% FBS but no growth factors. To maintain osmotic consistency across groups, 25 mM mannitol was added to the basal medium (which already contains 5 mM D-glucose) for the control condition, while 25 mM D-glucose was added on top of the basal medium for the HG condition. Cells were maintained under these conditions for 4 days, followed by exposure to either normoxia (21% oxygen) or Hyp (2% oxygen) with 5% carbon dioxide for an additional 24 h. After treatment, cells were collected for metabolomic profiling, proteomics, or transferred to Matrigel for the tube formation assay. A schematic overview of the experimental workflow is shown in Supplementary Figure S2.

### Harvesting HRECs for metabolomic analysis

Before harvesting HRECs for metabolomic analysis using Liquid Chromatography with tandem mass spectrometry (LC-MS/MS), the HRECs were rinsed thoroughly with warm PBS after removal of the culture media. Subsequently, while the HRECs were kept in the Petri dishes, liquid nitrogen was utilized to quench the metabolism of these cells rapidly. Afterward, each Petri dish was subjected to 1 mL of 80% pre-chilled methanol to quench the HREC metabolism to help extract intracellular metabolites. The HRECs were collected and carefully transferred into 1.5 mL centrifuge tubes using a sterile scraper. Finally, the collected cells were preserved at −80 °C until the start of further metabolomic analysis described below.

### Angiogenesis assay

The angiogenic ability of endothelial cells was assessed using a Matrigel-based assay, as previously published (Ibrahim et al., 2015). Briefly, HRECs treated under the above-described conditions were seeded onto 96-well plates pre-coated with Matrigel (Corning, Catalog #354234). Cells were plated in growth factor-depleted medium and incubated under their respective conditions for 18 h. Tube-like network formation was imaged using phase-contrast microscopy (Echo-Rebel), and the total tube length and number of branching points were quantified using Echo-Rebel’s image analysis tools.

### Proteomic analysis of HRECs

Proteomic analysis was performed at the Wayne State University Proteomics Core following established protocols as previously described (Scofield et al., 2025). In brief, HREC pellets were lysed in lithium dodecyl sulfate (LiDS) using a Bullet Blender for mechanical homogenization to ensure efficient protein extraction. After lysis, samples were heat-treated, clarified by filtration, and total protein concentrations were measured. The lysates were then reduced with dithiothreitol (DTT) and alkylated with iodoacetamide (IAA), followed by protein precipitation using phosphoric acid and methanol-based cleanup steps. The dried protein pellets were reconstituted in digestion buffer and subjected to overnight trypsin digestion at 37 °C. Peptides were then separated by liquid chromatography and analyzed using data-independent acquisition (DIA) mass spectrometry on an Orbitrap Eclipse system. Protein identification and data analysis were performed using Spectronaut software against the *Homo sapiens* UniProt database.

### Patient recruitment, surgery, and collection of samples

The Institutional Review Board of Wayne State University (IRB#: 090319MP2E) approved the collection of vitreous humor samples used in this study, which adhered to the principles outlined in the Declaration of Helsinki. Samples were obtained from patients with PDR, while the control group consisted of patients without clinical evidence of PDR, all of whom underwent pars plana vitrectomy (PPV) for conditions such as epiretinal membrane or macular hole. A vitreoretinal surgeon performed all vitrectomies. Undiluted vitreous samples (up to 1,000 μL) were collected at the start of the three-port PPV using manual aspiration into a syringe with the cutting function activated. All patients with vitreous humor samples collected during PPV provided written informed consent. Once collected, liquid nitrogen was used to freeze the vitreous humor samples and the samples were kept at −80 °C. Afterward, the vitreous samples were treated with 1 mL of 80% pre-chilled methanol to obtain metabolites, which underwent nucleotide analysis, as described below.

### LC-MS/MS metabolomic analysis

A LC-MS/MS-based metabolic approach was employed to quantitatively profile nucleotide-related metabolites in HRECs and vitreous fluid samples. Metabolite analysis was performed using the AB SCIEX QTRAP 6500 LC-MS/MS system, which combines a SHIMADZU Nexera ultra-high-performance liquid chromatography (UHPLC) unit with a triple quadrupole/linear ion trap mass spectrometer. Data acquisition was carried out using Analyst 1.6 software, while MultiQuant 3.0 software was utilized for data processing and quantitation. Metabolite extraction began with the addition of 1 mL of pre-chilled methanol (−80 °C) to harvested HRECs or 200 µL of vitreous fluid, followed by vortexing and centrifugation at 10,000 rpm for 10 min at 4 °C. The supernatant was dried using a CentriVap Refrigerated Concentrator (10 °C) and reconstituted in a 50:50 (v/v) acetonitrile-water mixture. After vortexing and centrifugation, the supernatant was diluted and analyzed using two LC-MS/MS runs: reverse-phase liquid chromatography and hydrophilic-interaction liquid chromatography, as previously described (Gregory et al., 2023; Bao et al., 2019). For each individual nucleotide, a calibration standard was prepared in a range of 10 nM to 10 μM using the appropriate mobile phase.

### Data analysis

Metabolomic data were assessed for normality and log-normality using the Shapiro–Wilk test. Because group sample sizes were equal, ANOVA is generally robust to minor deviations from normality; therefore, when data passed or showed only minor deviations (e.g., one group failing), one-way ANOVA was applied, followed by Fisher’s *post hoc* test, with statistical significance set at *p* < 0.05. In cases of major deviation from normality (e.g., more than two groups failing the test), the non-parametric Kruskal–Wallis test was applied. The specific statistical test used is indicated in the figure legends. Proteomic data were analyzed using two-way ANOVA with Fisher’s *post hoc* test for pairwise group comparisons. Principal component analysis (PCA) of detected nucleotides was performed using the R package ggbiplot to visualize group separation. Vitreous sample data were analyzed using two-tailed *t*-tests between PDR and non-PDR groups, and receiver operating characteristic (ROC) curve analyses, as well as odds ratio calculations were performed using MedCalc statistical software.

## Results

### Profiles of Nucleotide-related metabolites in HRECs exposed to HG and Hyp

**FIGURE 1:**
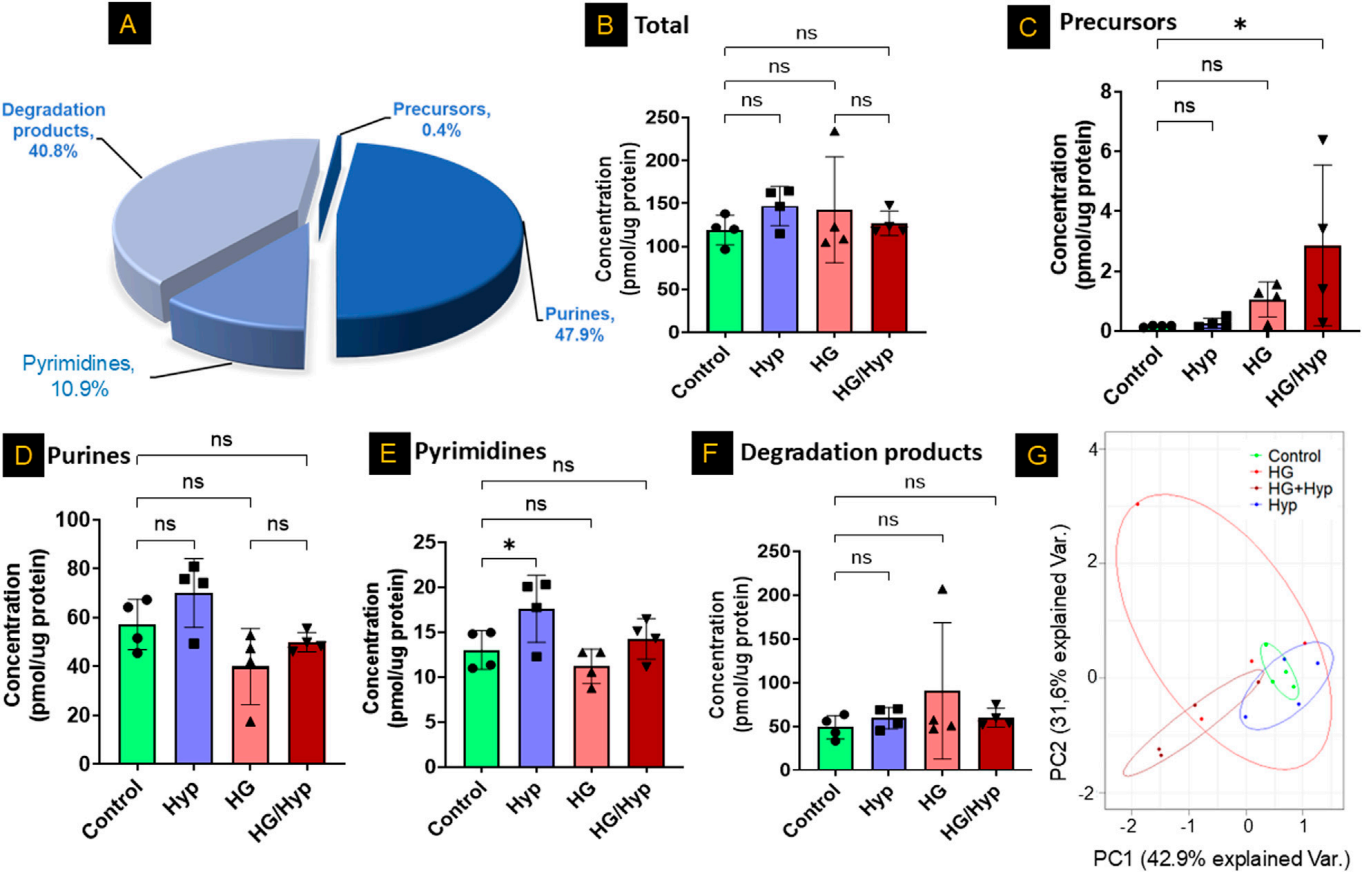
Profiles of Nucleotide-Related Metabolites in Human Retinal Endothelial Cells (HRECs) Exposed to High Glucose (HG) and Hypoxia (Hyp). **(A)** Representative nucleotide profile in HRECs derived from healthy donors and analyzed by LC-MS/MS. Quantification of nucleotide-related metabolites in **(B)** total pool, **(C)** nucleotide precursors, **(D)** purines, **(E)** pyrimidines, and **(F)** nucleotide degradation products across the four experimental groups: control, HG, Hyp, and HG+Hyp. **(G)** Principal component analysis (PCA) of overall nucleotide-related metabolites showing group separation. HRECs were cultured in basal medium (5 mM D-glucose, 5% FBS, without growth factors). For osmotic control, 25 mM mannitol was added; for HG treatment, 25 mM D-glucose was added. Cells were treated for 4 days, then exposed to normoxia (21% O_2_) or Hyp (2% O_2_, 5% CO_2_) for 24 h prior to analysis. Data are shown as mean ± SD; n = 4 per group. ns = not significant; *P < 0.05 by one-way ANOVA with Fisher’s *post hoc* test.

We first analyzed the nucleotide profile of HRECs derived from healthy donors and cultured under control conditions using LC-MS/MS. As shown in Figure 1A, the nucleotide pool consisted of purine (∼48%) and pyrimidine (∼11%) metabolites, along with their degradation products (∼41%) and biosynthetic precursors (∼0.5%). We then assessed the nucleotide profile of HRECs exposed to HG and Hyp, both of which are risk factors for PDR and known to induce the Warburg effect. The induction of the Warburg effect under these conditions was validated in our prior study (Gregory et al., 2023), which demonstrated increased glucose uptake and lactate production in HRECs exposed to HG, Hyp, or their combination. In the current study, pathway enrichment analysis further supported activation of the Warburg effect under HG+Hyp (Supplementary Figure S1), which was confirmed by proteomic analysis showing significant upregulation of glucose transporter (GLUT)1 and lactate dehydrogenase (LDH) (Supplementary Figure S3A). Importantly, neither HG nor Hyp compromised endothelial cell viability, as assessed by LDH release. Relative viability values (mean ± SD) were 1.00 ± 0.14 for control, 0.85 ± 0.12 for Hyp, 1.07 ± 0.21 for HG, and 1.21 ± 0.31 for HG+Hyp, with no significant differences compared to control. Elevated GLUT1 and LDH levels are therefore consistent with enhanced glucose uptake and lactate production, hallmark features of Warburg metabolism, and not a result of cytotoxicity.

Next, we employed LC-MS/MS to assess the total concentrations of nucleotide-related metabolites across different treatment groups, including HG alone, Hyp alone, HG+Hyp, and control conditions. Surprisingly, no significant global differences were observed in total nucleotide-related metabolite levels among these groups (Figure 1B). This finding remained consistent when metabolites were stratified into major classes, with no significant changes in total purines, total pyrimidines, or total degradation products (Figures 1D–F). In contrast, nucleotide precursors exhibited significant alterations (Figure 1C). Principal component analysis further revealed a partial separation of nucleotide-related metabolite profiles across HG, Hyp, HG+Hyp, and control groups (Figure 1G), prompting a more detailed investigation into the specific metabolic changes driving this separation.

### Analysis of nucleotide precursors in HRECs exposed to HG and Hyp

**FIGURE 2:**
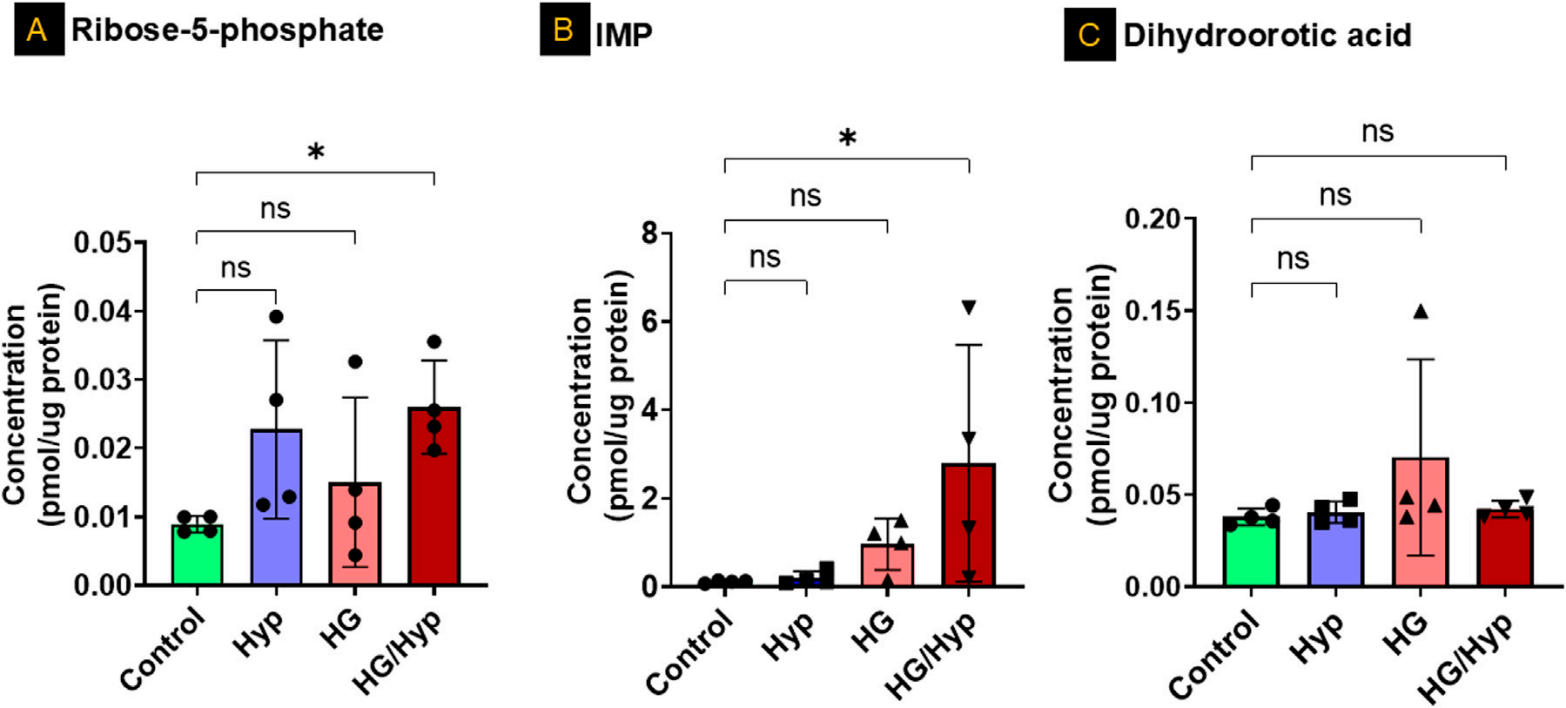
Analysis of Nucleotide Precursors in HRECs Exposed to HG and Hyp. Concentrations of **(A)** ribose-5-phosphate, **(B)** inosine monophosphate (IMP), and **(C)** dihydroorotic acid in HRECs. HRECs were maintained in basal medium containing 5 mM D-glucose and 5% FBS without growth factors. For osmotic control, 25 mM mannitol was added; for HG treatment, 25 mM D-glucose was added. Cells were treated for 4 days, then exposed to normoxia (21% O_2_) or Hyp (2% O_2_, 5% CO_2_) for 24 h prior to analysis. Data are shown as mean ± SD; n = 4 per group. ns = not significant; *P < 0.05 by one-way ANOVA with Fisher’s *post hoc* test.

To further investigate the separation observed among the groups in the principal component analysis (Figure 1G) and determine whether specific nucleotides exhibited significant differences in concentration between treatment conditions, we first analyzed purine and pyrimidine precursors in HRECs exposed to HG and Hyp, compared to either stressor alone. The analysis showed significant differences in the concentrations of D-ribose-5-phosphate, a glycolytic precursor to both pyrimidines and purines, as well as in the levels of inosine monophosphate (IMP), a precursor to purines, in the HG+Hyp group compared to the control group (Figures 2A,B, respectively). D-ribose-5-phosphate and IMP levels remained unchanged in Hyp or HG alone compared with the control. On the other hand, no significant differences in the concentration of dihydroorotic acid, a pyrimidine precursor, were observed among all experimental groups (Figure 2C). These results demonstrate the distinct impact of HG+Hyp on the metabolism of specific nucleotide precursors, compared to the effects of HG or Hyp alone.

### Analysis of purine and pyrimidine nucleotides in HRECs exposed to HG and Hyp

**FIGURE 3:**
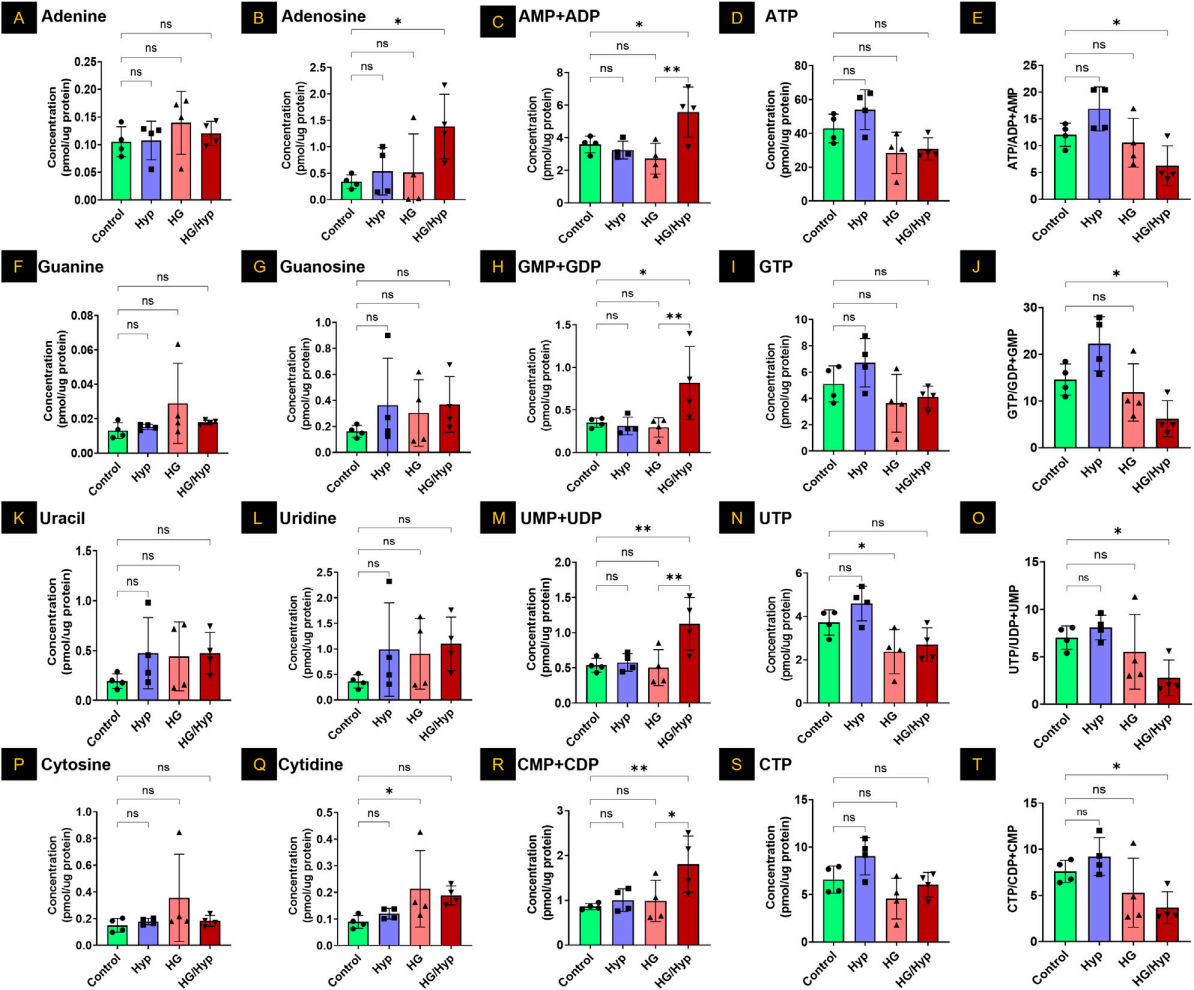
Analysis of Purine and Pyrimidine Nucleotides in HRECs Exposed to HG and Hyp. Concentrations of **(A)** adenine, **(B)** adenosine, **(C)** combined adenosine monophosphate (AMP) and adenosine diphosphate (ADP), **(D)** adenosine triphosphate (ATP), **(E)** ATP/AMP + ADP ratio, **(F)** guanine, **(G)** guanosine, **(H)** guanosine monophosphate (GMP) and guanosine diphosphate (GDP) combined, **(I)** guanosine triphosphate (GTP), **(J)** GTP/GMP+GDP ratio, **(K)** uracil, **(L)** uridine, **(M)** uridine monophosphate (UMP) and uridine diphosphate (UDP) combined, **(N)** uridine triphosphate (UTP), **(O)** UTP/UMP + UDP ratio, **(P)** cytosine, **(Q)** cytidine, **(R)** cytidine monophosphate (CMP) and cytidine diphosphate (CDP) combined, **(S)** cytidine triphosphate (CTP), and **(T)** CTP/CMP+CDP ratio in HRECs. Cells were cultured in basal medium (5 mM D-glucose, 5% FBS, without growth factors). For osmotic control, 25 mM mannitol was added; for HG treatment, 25 mM D-glucose was added. Cells were treated for 4 days, then exposed to normoxia (21% O_2_) or Hyp (2% O_2_, 5% CO_2_) for 24 h prior to analysis. Data are shown as mean ± SD; n = 4 per group. ns = not significant; *: P < 0.05; **: P < 0.01 by one-way ANOVA with Fisher’s *post hoc* test.

Subsequently, adenine and its associated metabolites were analyzed to assess changes in their concentrations under conditions of HG and/or Hyp. Concerning the simple nucleotide base adenine, no significant differences between HG, Hyp, or combined HG/Hyp were noted relative to the control group (Figure 3A). However, adenosine, a direct precursor to adenosine phosphate species, showed a threefold elevation in HRECs exposed to the combined effects of HG and Hyp compared to control (Figure 3B). A similar trend was observed in Figure 3C when assessing the pooled levels of adenosine monophosphate (AMP) and adenosine diphosphate (ADP). The AMP+ADP pool exhibited a twofold increase in HRECs exposed to the combined effects of HG and Hyp compared to the control. Interestingly, while the levels of energy-abundant adenosine triphosphate (ATP) showed only a slight decrease under the combined effects of HG and Hyp compared to the control group (Figure 3D), the ATP to AMP+ADP ratio was significantly reduced, being approximately half that of the control group (p-value <0.05, Figure 3E).

When considering the other purine base, guanine, no significant differences were observed among HRECs cultured under conditions of HG and/or Hyp (Figure 3F). Similarly, the levels of the ribose sugar guanosine were consistent across the control, Hyp, HG, and HG/Hyp exposures (Figure 3G). However, when assessing the levels of guanosine monophosphate (GMP) and guanosine diphosphate (GDP) pool, a nearly twofold increase was observed in HRECs exposed to the combined effects of HG and Hyp compared to the control (p-value <0.05, Figure 3H). Additionally, similar to the triphosphate analog of adenosine, guanosine triphosphate (GTP) levels showed a slight decrease under HG+Hyp relative to control (Figure 3I). However, the ratio of GTP to GMP+GDP was significantly reduced to half under the HG+Hyp condition compared to the control (Figure 3J).

Consistent with the observations in purine bases, uracil levels were slightly, though not significantly, elevated in HRECs under HG+Hyp compared to the control (Figure 3K). Similarly, uridine concentrations, a ribose sugar analog, showed no significant difference between control and HG+Hyp conditions (Figure 3L). However, when HRECs were subjected to the combined treatment of HG and Hyp, the cells exhibited a significant twofold increase in the levels of uridine monophosphate (UMP) and uridine diphosphate (UDP) pools (Figure 3M) and a slight decrease in the levels of uridine triphosphate (UTP) pools (Figure 3N). Following the trends observed in the purine triphosphate to mono- and di-phosphate ratios, the UTP to UMP+UDP ratio under the dual stress of HG+Hyp was half of that in the control condition, p-value <0.05 (Figure 3O).

Similar to uracil, the other pyrimidine base, cytosine, showed no significant change in concentration in HRECs exposed to HG and/or Hyp compared with controls (Figure 3P). However, its ribose sugar, cytidine, had levels approximately twofold higher under the effect of HG in the presence or absence of Hyp compared to the control condition (Figure 3Q). Additionally, when assessing the monophosphate (CMP) and diphosphate (CDP) pool of this nucleotide, there was a significant twofold increase in the levels of this pool only when HRECs were subjected to the combined treatment of HG and Hyp compared to the control (Figure 3R). The levels of cytidine triphosphate (CTP) were not statistically significantly different in HRECs treated with HG and/or Hyp compared to the control group (Figure 3S). Following the trends observed in the purines and uracil, the CTP to CMP+CDP ratio under HG+Hyp was half of that in the control condition (p-value <0.05) (Figure 3T).

### The profile of nucleotide degradation products in HRECs exposed to HG and Hyp

**FIGURE 4:**
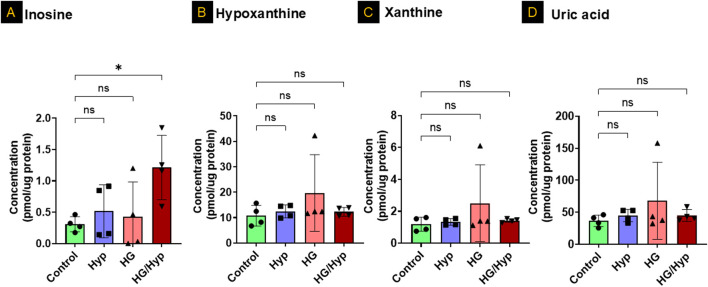
The Profile of Nucleotide Degradation Products in HRECs Exposed to HG and Hyp. Concentrations of **(A)** inosine, **(B)** hypoxanthine, **(C)** xanthine, and **(D)** uric acid in HRECs. Cells were maintained in basal medium containing 5 mM D-glucose and 5% FBS without growth factors. For osmotic control, 25 mM mannitol was added; for HG treatment, 25 mM D-glucose was added. Cells were treated for 4 days, then exposed to normoxia (21% O_2_) or Hyp (2% O_2_, 5% CO_2_) for 24 h prior to analysis. Data are shown as mean ± SD; n = 4 per group. ns = not significant; *P < 0.05 by one-way ANOVA with Fisher’s *post hoc* test.

Figure 4 presents the levels of purine degradation products, including inosine, hypoxanthine, xanthine, and uric acid, in HRECs subjected to HG, Hyp, and their combination. As shown in Figure 4A, inosine exhibited a significant threefold increase in its concentration when HRECs were subjected to the combined treatment of HG and Hyp compared to the control group, while its levels remained similar when HRECs were treated with either HG or Hyp alone. In contrast, no significant differences were observed in the concentrations of other nucleotide degradation products, including hypoxanthine, xanthine, and uric acid, among all experimental groups (Figures 4B–D, respectively). These results indicate a differential impact of HG+Hyp, compared to HG or Hyp alone, on purine metabolism, specifically altering inosine levels without affecting the concentrations of other purine catabolic products.

### Enriched pathway analysis of nucleotides in HRECs under the combined effect of HG+Hyp

**FIGURE 5:**
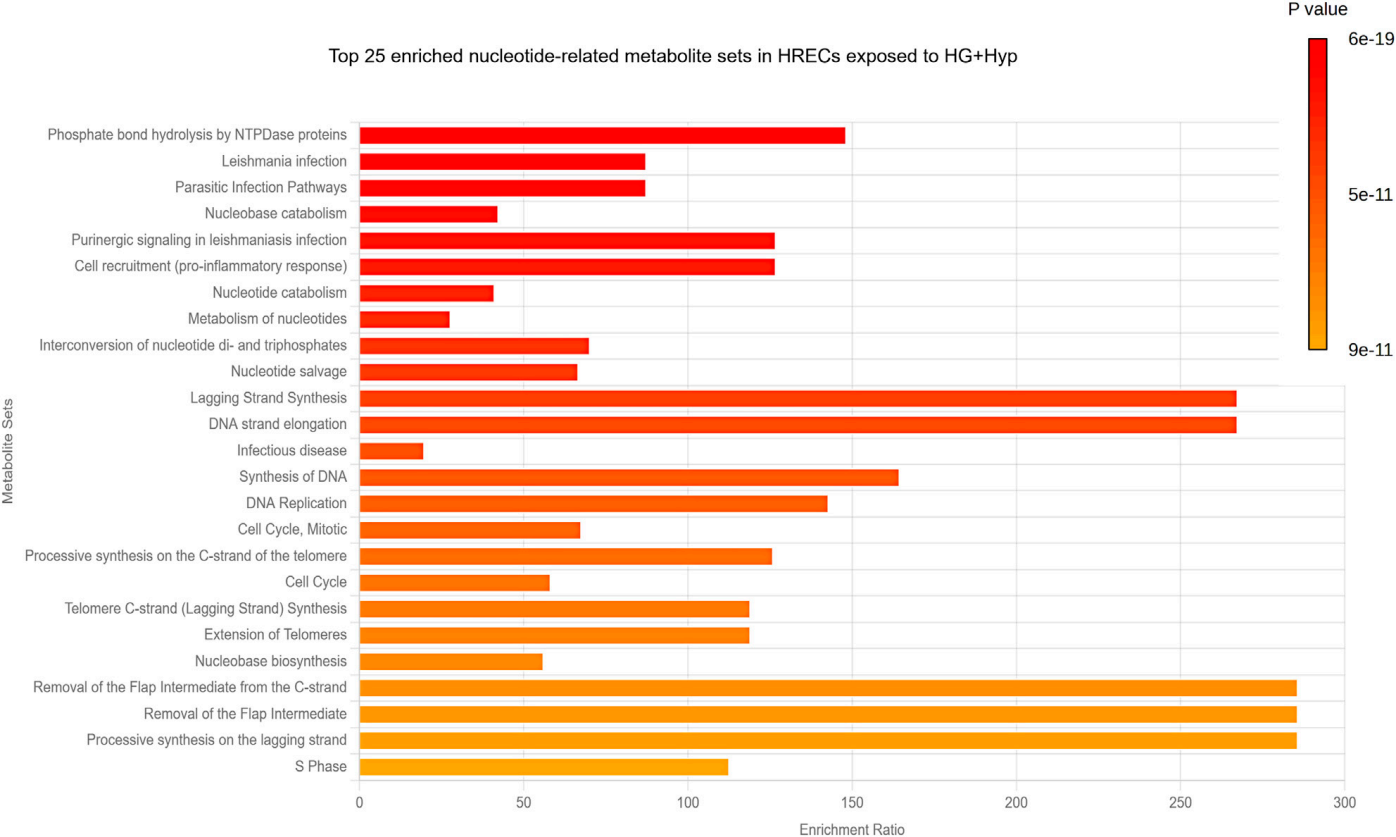
Pathway Analysis of Nucleotide-Related Metabolites in HRECs Exposed to HG and Hyp. Significantly altered purine- and pyrimidine-related metabolites in HRECs under HG and Hyp were analyzed against curated pathways from the Relational Database of Metabolomics Pathways (RaMP-DB) to identify the top 25 key metabolic pathways impacted.

Next, a pathway analysis was performed using the list of significantly altered metabolites against curated pathways from the Relational Database of Metabolomics Pathways (RaMP-DB), which integrates data from the Kyoto Encyclopedia of Genes and Genomes (KEGG), the Human Metabolome Database (HMDB), Reactome, and WikiPathways. This analysis revealed significant enrichment (FDR <0.05) in pathways primarily related to nucleotide metabolism and DNA synthesis/replication processes (Figure 5). The most significantly enriched pathways included phosphate bond hydrolysis by NTPDase proteins, nucleobase and nucleotide catabolism, nucleotide salvage, interconversion of nucleotide di- and triphosphates, and nucleotide metabolism. In addition, pathways associated with DNA strand elongation, lagging-strand synthesis, telomere extension, and cell cycle regulation were significantly enriched, underscoring the impact of HG and Hyp-induced metabolic reprogramming on DNA synthesis and endothelial cell proliferation. Interestingly, several infection- and inflammation-related pathways (e.g., Leishmania infection, parasitic infection, pro-inflammatory cell recruitment) also appeared enriched. These results are likely attributable to overlapping nucleotide turnover and purinergic signaling components that are shared across host-pathogen, immune response, and DNA synthesis pathways.

Taken together, these findings suggest that the combined effect of HG and Hyp impacts several pathways involved in the progression of endothelial cell proliferation and angiogenesis. To validate these pathways, an *in vitro* angiogenesis assay was performed using the Matrigel-based tube formation assay. The results showed that the HG+Hyp condition significantly enhanced angiogenic activity, as evidenced by a marked increase in total tube length of HRECs compared to the control, HG alone, or Hyp alone conditions (Supplementary Figures S3B,C).

### Proteomic profiling reveals metabolic rewiring underlying nucleoside mono- and diphosphate accumulation in HRECs exposed to HG+Hyp

**FIGURE 6:**
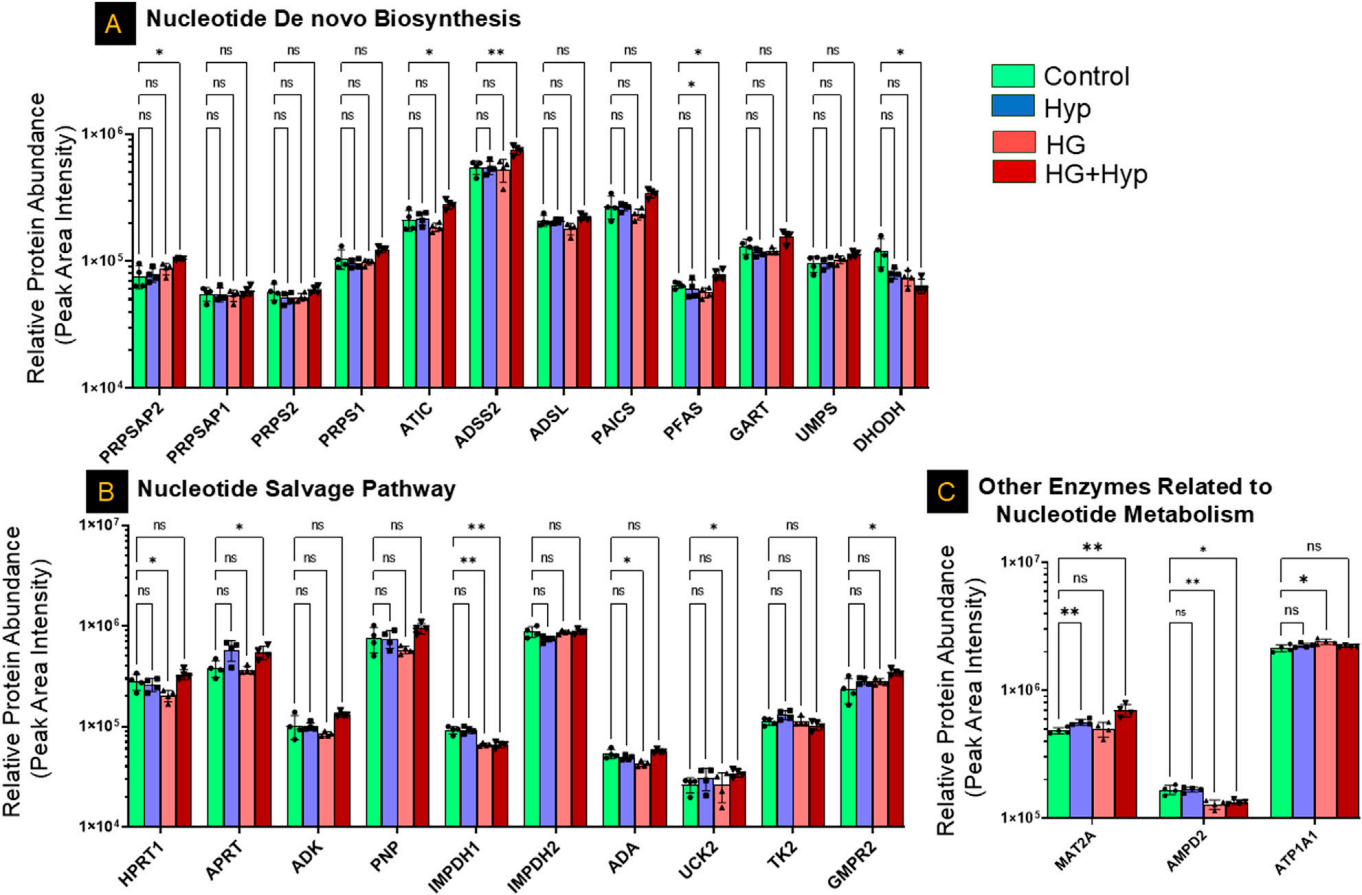
Proteomic Profiling of Nucleotide Metabolism-Related Enzymes in HRECs under the Effect of HG and Hyp. Relative protein abundance (based on peak area intensity) of enzymes involved in **(A)** *de novo* nucleotide biosynthesis, including phosphoribosyl pyrophosphate synthetase-associated protein 2 (PRPSAP2), PRPSAP1, phosphoribosyl pyrophosphate synthetase 1 (PRPS1), PRPS2, 5-aminoimidazole-4-carboxamide ribonucleotide formyltransferase/IMP cyclohydrolase (ATIC), adenylosuccinate synthase 2 (ADSS2), adenylosuccinate lyase (ADSL), phosphoribosylaminoimidazole succinocarboxamide synthetase (PAICS), phosphoribosylformylglycinamidine synthase (PFAS), phosphoribosylglycinamide formyltransferase (GART), uridine monophosphate synthetase (UMPS), and dihydroorotate dehydrogenase (DHODH); **(B)** nucleotide salvage pathway, including hypoxanthine-guanine phosphoribosyltransferase (HPRT1), adenine phospho-ribosyltransferase (APRT), adenosine kinase (ADK), purine nucleoside phosphorylase (PNP), inosine-5′-monophosphate dehydrogenase 1 and 2 (IMPDH1, IMPDH2), adenosine deaminase (ADA), uridine-cytidine kinase 2 (UCK2), thymidine kinase 2 (TK2), and guanosine monophosphate reductase 2 (GMPR2); and **(C)** other nucleotide metabolism-related enzymes, including methionine adenosyltransferase II alpha (MAT2A), adenosine monophosphate deaminase 2 (AMPD2), and ATPase subunit alpha 1 (ATP1A1). HRECs were initially cultured in complete growth medium containing 5 mM D-glucose, 5% FBS, and growth factors. For experimental treatments, cells were switched to basal medium containing 5 mM D-glucose and 5% FBS but without growth factors. To maintain osmotic balance, 25 mM mannitol was added for the control group, while 25 mM D-glucose was added for the HG group. Cells were treated for 4 days, followed by exposure to either normoxia (21% O_2_) or Hyp (2% O_2_, 5% CO_2_) for an additional 24 h prior to analysis. Bars represent mean ± SD; n = 4 per group. *p < 0.05, **p < 0.01; ns: not significant. Proteomic data were analyzed using two-way ANOVA with Fisher’s *post hoc* test applied for pairwise group comparisons.

Analysis of nucleotide metabolism in HRECs cultured under HG+Hyp revealed selective alterations in both biosynthetic and salvage pathways, rather than a global shift in nucleotide homeostasis. Proteomic analysis identified significant upregulation of several key enzymes involved in nucleotide *de novo* biosynthesis, including phosphoribosyl pyrophosphate synthetase-associated protein (PRPSAP)2, phosphoribosylformylglycinamidine synthase (PFAS), adenylosuccinate synthase (ADSS)2, and AICAR transformylase/IMP cyclohydrolase (ATIC). In contrast, dihydroorotate dehydrogenase (DHODH), a critical enzyme in the pyrimidine *de novo* pathway, was significantly downregulated under HG+Hyp conditions. Other components of the *de novo* pathway, including PRPSAP1, PRPS1, PRPS2, ADSL, PAICS, GART, and UMPS, remained unchanged between the HG+Hyp and control groups (Figure 6A).

In the nucleotide salvage pathway, significant upregulation of adenine phosphoribosyltransferase (APRT), uridine-cytidine kinase (UCK)2, and guanosine monophosphate reductase (GMPR)2 was observed under HG+Hyp conditions, alongside a significant downregulation of inosine-5′-monophosphate dehydrogenase (IMPDH)1. However, the expression of hypoxanthine-guanine phosphoribosyltransferase (HPRT)1, adenosine kinase (ADK), purine nucleoside phosphorylase (PNP), IMPDH2, adenosine deaminase (ADA), and thymidine kinase (TK)2 did not differ significantly between HG+Hyp and control groups (Figure 6B). Among other enzymes implicated in nucleotide metabolism, methionine adenosyltransferase II alpha (MAT2A) was significantly upregulated under HG+Hyp, while adenosine monophosphate deaminase (AMPD)2 was significantly downregulated, suggesting an imbalance in AMP production and degradation. No significant change was noted in ATPase Na+/K+ transporting subunit alpha (ATP1A)1 (Figure 6C).

**FIGURE 7:**
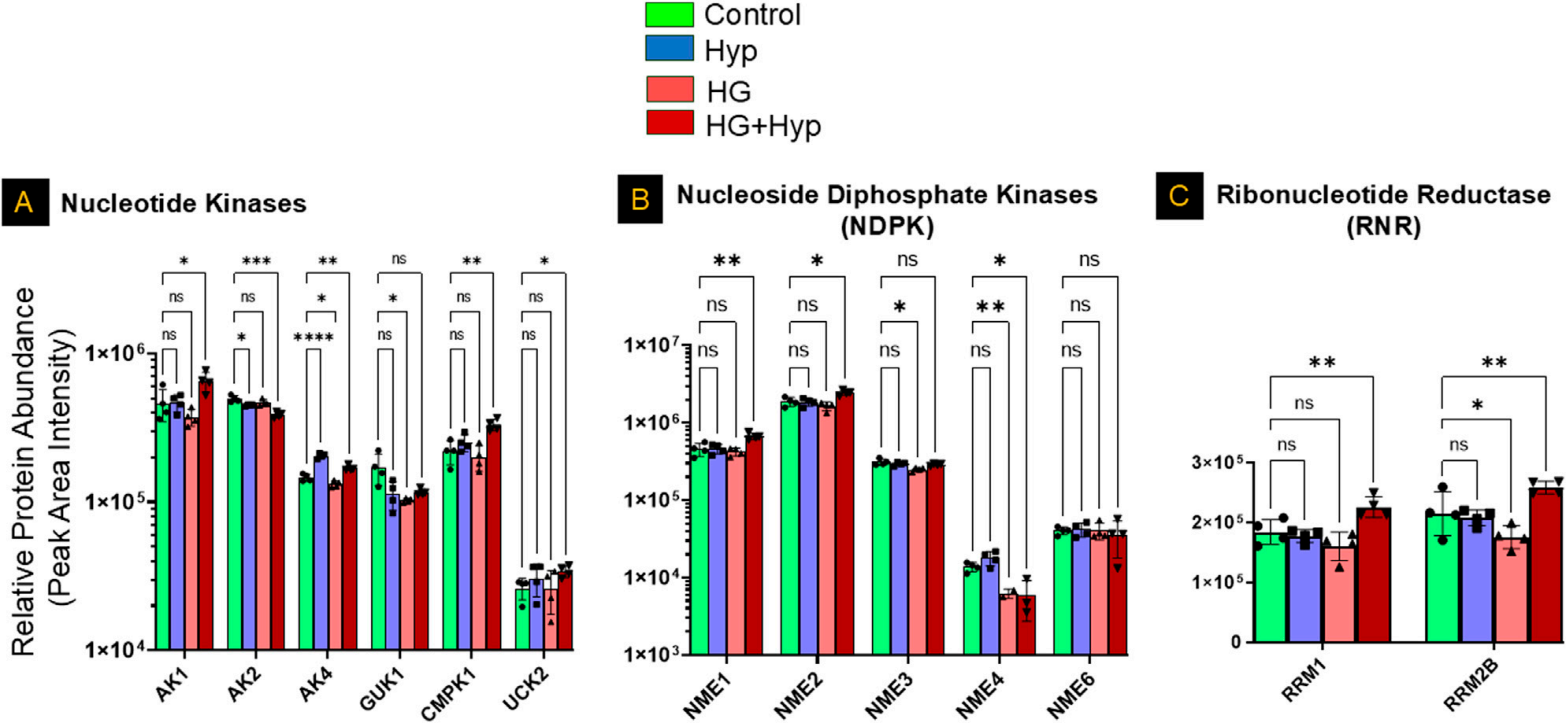
Proteomic Profiling of Nucleotide Kinases, Nucleoside Diphosphate Kinases, and Ribonucleotide Reductases in HRECs Exposed to HG and Hyp. Relative protein abundance (based on peak area intensity) of **(A)** nucleotide kinases, including adenylate kinase 1 (AK1), AK2, AK4, cytidylate monophosphate kinase 1 (CMPK1), and uridine-cytidine kinase 2 (UCK2); **(B)** nucleoside diphosphate kinases (NME family), including NME1, NME2, NME3, NME4, and NME6; and **(C)** ribonucleotide reductase subunits, including ribonucleotide reductase subunit M1 (RRM1) and subunit M2B (RRM2B). HRECs were initially cultured in complete growth medium containing 5 mM D-glucose, 5% fetal bovine serum (FBS), and growth factors. For experimental treatments, cells were switched to basal medium containing 5 mM D-glucose and 5% FBS but without growth factors. To maintain osmotic balance, 25 mM mannitol was added for the control group, while 25 mM D-glucose was added for the high glucose (HG) group. Cells were treated for 4 days, followed by exposure to either normoxia (21% O_2_) or Hyp (2% O_2_, 5% CO_2_) for an additional 24 h prior to analysis. Following treatment, cells were collected and processed for proteomic profiling using LC-MS/MS. Data are presented as relative protein abundance (peak area intensity). Bars represent mean ± SD (n = 4 per group). Statistical comparisons were performed between the control and each treatment group. *p < 0.05, **p < 0.01, and ***p < 0.001; ns: not significant. Proteomic data were analyzed using two-way ANOVA with Fisher’s *post hoc* test applied for pairwise group comparisons.

Further analysis of nucleotide kinases demonstrated significant upregulation of adenylate kinase (AK)1, AK4, UCK2, and cytidylate monophosphate kinase (CMPK)1 under HG+Hyp, whereas mitochondrial adenylate kinase (AK)2 was significantly downregulated. Guanylate kinase (GUK)1 remained unchanged. These observations align with the nucleotide shifts detected at the metabolite level, suggesting a dysregulated nucleotide turnover and energy stress response (Figure 7A).

Consistent with this, the nucleoside diphosphate kinase (NME) family showed significant isoform-specific alterations. Both NME1 and NME2, the cytosolic isoforms, were significantly upregulated, whereas the mitochondrial isoform NME4 was significantly downregulated under HG+Hyp conditions. No significant differences were observed in NME3 or NME6 levels (Figure 7B). Analysis of ribonucleotide reductase subunits further revealed significant upregulation of both RRM1 and RRM2B in the HG+Hyp group, supporting the notion of an increased demand for deoxynucleotides in response to metabolic and proliferative stress induced by the Warburg effect (Figure 7C).

Together, these findings indicate that HRECs exposed to HG and Hyp display coordinated metabolic rewiring across *de novo* synthesis, salvage, and nucleotide conversion pathways. This metabolic signature is characterized by enhanced nucleotide precursor production and altered kinase expression, favoring the accumulation of NMPs and NDPs over their triphosphate counterparts.

### Mono- and diphosphate nucleotide levels in the vitreous of patients with PDR

**FIGURE 8:**
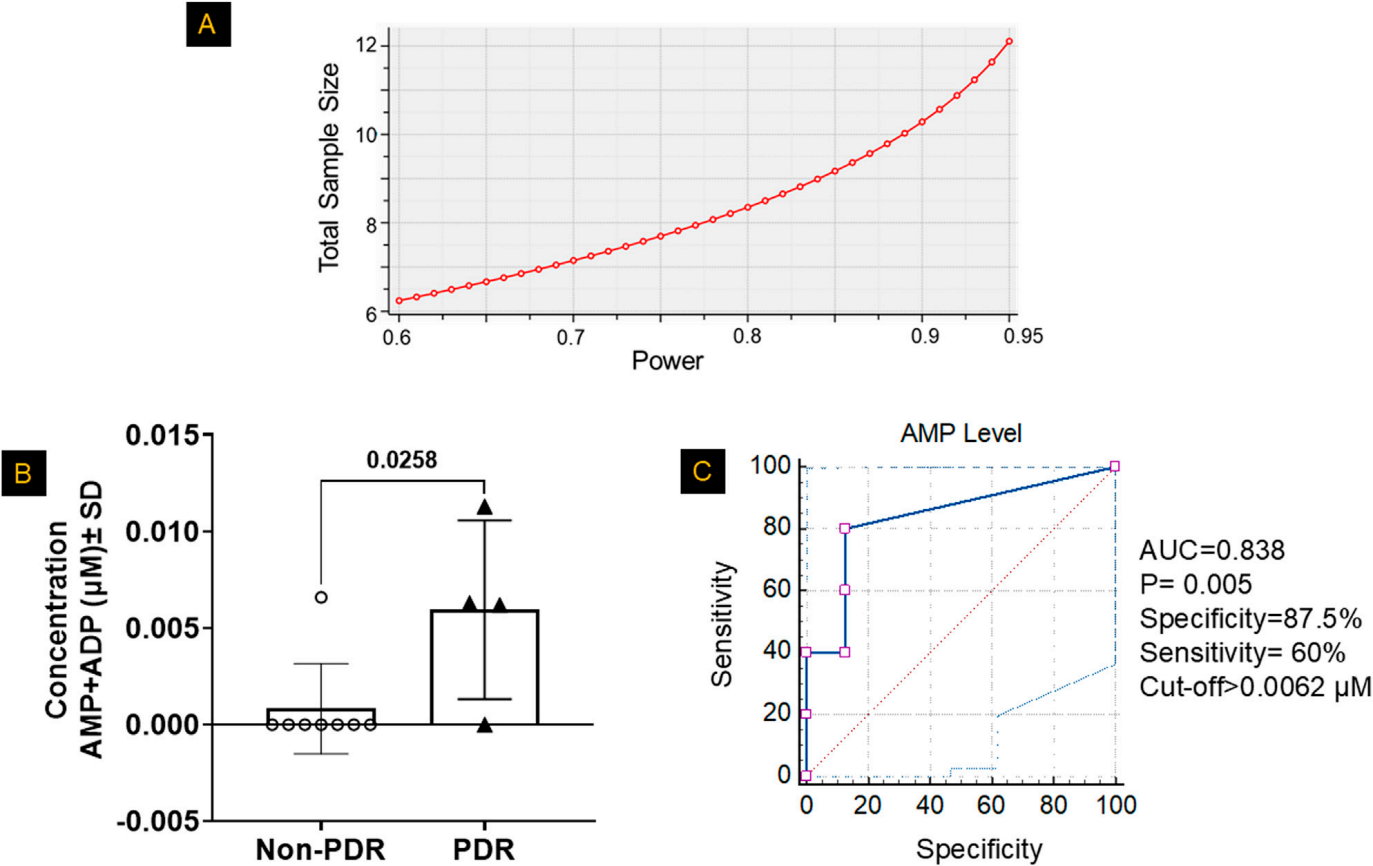
Mono- and Diphosphate Nucleotide Levels in the Vitreous of Patients with PDR. **(A)** Based on the power analysis from Figure 3C, the sample size calculation indicated that a sample size of 4-6 patients per group would provide ∼80–95% power to detect significant differences. **(B)** LC-MS/MS analysis revealed significantly higher combined levels of adenosine monophosphate (AMP) and adenosine diphosphate (ADP) in the vitreous humor of patients with proliferative diabetic retinopathy (PDR) compared to non-PDR controls, after excluding outliers identified by Grubbs’ test. Data are presented as mean ± SD; n = 4–8 per group. The comparison between the two groups was performed using unpaired two-tailed t-test. **(C)** A receiver operating characteristic (ROC) curve analysis was used to determine the optimal cutoff value for classifying patients based on high or low AMP+ADP levels, evaluating the diagnostic performance of AMP+ADP as a predictor.

To determine whether the nucleotide alterations observed in HRECs under HG+Hyp conditions are reflected *in vivo*, we analyzed mono- and diphosphate nucleotide levels in vitreous samples from patients with PDR and non-PDR controls. The vitreous was selected because it directly bathes areas of retinal neovascularization and serves as a proximal biofluid reflecting the metabolic state of retinal endothelial cells. We further sought to evaluate the relationship between vitreous mono- and diphosphate nucleotide levels and PDR for potential translational relevance. To this end, we measured nucleotide concentrations in vitreous samples obtained from patients with PDR and from non-PDR controls undergoing PPV for epiretinal membrane or macular hole. Demographic and clinical characteristics of the patient cohorts are summarized as follows. The non-PDR group included 8 patients (5 males, 3 females) with a mean age of 70.1 ± 9.7 years. The PDR group included 5 patients (4 males, 1 female) with a mean age of 59.1 ± 13.0 years. None of the patients had received prior anti-VEGF therapy. There were no significant differences in age (p = 0.165, t-test) or gender distribution (p = 1.0, Fisher’s exact test) between the non-PDR and PDR groups. Interestingly, guanylate, cytidylate, and uridylate mono- and diphosphate nucleotide levels were undetectable in PDR samples, with only the adenylated pool being detectable (data not shown). Consequently, the sample size was determined using a power analysis based on the AMP+ADP pool data from Figure 3C, which showed a significant increase in AMP+ADP levels in HRECs under HG+Hyp (5.6 ± 1.5 pmol/μg protein) compared to the control group (3.6 ± 0.5 pmol/μg protein). The power analysis was conducted using G*Power 3.1.9.4 software with an *a priori* analysis approach, a significance level (α) of 0.05 (two-tailed), and a desired power (1-β) of 80%–95%. The expected effect size (d) was calculated from the mean and standard deviation of each group. As shown in Figure 8A, this analysis determined that a sample size of 8–12 participants (4-6 per group) was sufficient to achieve the desired statistical power. With this sample size, subsequent LC-MS/MS analysis of the vitreous samples in Figure 8B showed that the PDR group had significantly higher levels of the AMP+ADP pool (0.006 µM ± 0.004 SD) compared to the non-PDR group (0.0008 µM ± 0.002 SD; p < 0.05). One patient from the PDR cohort was excluded from the metabolomics analysis because the combined AMP+ADP level was identified as a positive outlier by the Grubbs’ test.

After confirming a significant increase in the AMP+ADP pool levels in PDR patients, we aimed to assess the strength of the association between the AMP+ADP pool and PDR. To achieve this, we calculated odds ratios by comparing the likelihood of having high AMP+ADP pool levels in PDR cases versus non-PDR controls. The samples were categorized into high or low AMP+ADP pool levels using a predefined cutoff, determined via receiver operating characteristic (ROC) curves, which evaluated the distribution of the AMP+ADP pool in both PDR and control vitreous samples. The ROC analysis produced an area under the curve (AUC) of 0.838 (SE = 0.121, P = 0.005, 95% CI: 0.536–0.978). The optimal cutoff value for distinguishing PDR patients based on AMP+ADP pool concentration was found to be 0.0062 µM, balancing sensitivity and specificity (Figure 8C). At this threshold, specificity was 87.5%, and the odds of having PDR were significantly higher compared to non-PDR controls, indicating its potential as a diagnostic marker (Supplementary Table S1).

## Discussion

The primary finding of this study is that there is no widespread alteration in global nucleotide metabolism in HRECs experiencing the Warburg effect driven by PDR-related stressors, specifically HG and Hyp. However, a specific subset of nucleotides, namely nucleoside monophosphates (NMPs) and diphosphates (NDPs), are selectively affected. Under the combined effects of HG and Hyp, increased levels of NMPs and NDPs were observed across all four nucleotides when compared to their corresponding triphosphates (NTPs). This is supported by the observation that the AMP+ADP pool is significantly higher in vitreous samples from patients with PDR compared to those without PDR. Moreover, there is a strong association between high vitreous AMP+ADP levels and PDR, as indicated by a high odds ratio, suggesting that this accumulation may represent a hallmark of proliferative retinal pathology rather than a mere byproduct of the disease.

These findings are consistent with prior reports. Zeiner et al. (Zeiner et al., 2019) documented elevated ADP levels in vitreous samples from PDR patients (N = 48), while Loukovaara et al. (Loukovaara et al., 2015) reported increases in the combined AMP+ADP pool (N = 24). Our results confirm and expand upon these findings by introducing a diagnostic dimension: using ROC analysis, we established a detection threshold of 0.0062 µM for AMP+ADP, which yields a specificity of 87.5% for identifying PDR cases. These findings support the utility of AMP+ADP as a potential biomarker for PDR.

To gain insight into the mechanisms driving this selective nucleotide accumulation, we modeled the PDR microenvironment in HRECs by exposing the cells to HG and Hyp, the two major stressors associated with PDR. Under this combined condition, not only elevated AMP+ADP levels were seen, mirroring the vitreous phenotype in PDR patients, but also broader accumulations of GMP+GDP, CMP+CDP, and UMP+UDP, indicating global dysregulation of both NMP+NDP pools. To determine whether this response was specific to the combined condition or could also be triggered by either stressor alone, we compared HG, Hyp, and HG+Hyp exposures. While all three conditions have been shown to induce hallmark features of the Warburg effect, including increased glucose uptake and lactate production (Gregory et al., 2023), only the combined HG+Hyp condition redirected glycolytic intermediates toward NMP+NDP accumulation and enhanced angiogenic activity, as evidenced by increased tube formation (Supplementary Figures S3B,C). This subtype of the Warburg effect, induced by HG and Hyp, appears to promote the accumulation of NMPs and NDPs to meet the increased demand for deoxynucleotides needed for nucleic acid synthesis during endothelial proliferation and pathological angiogenesis, hallmarks of disease progression in PDR.

The accumulation of NMPs and NDPs under the Warburg effect induced by HG and Hyp can be attributed to several interconnected metabolic disturbances identified in our proteomic analysis (Figures 6, 7). A key contributor is impaired mitochondrial nucleotide metabolism, as evidenced by the significant downregulation of the mitochondrial nucleoside diphosphate kinase isoform NME4, which plays a crucial role in maintaining cellular NTP levels by converting NDPs to NTPs within the mitochondria (Wang et al., 2019). Notably, while cytosolic NDPK isoforms NME1 and NME2 were upregulated, possibly as a compensatory response, the continued accumulation of NDPs, despite this upregulation, suggests that mitochondrial NME4 plays a dominant role in sustaining nucleotide homeostasis under HG+Hyp conditions. Given that NTPs are essential for endothelial energy transfer, signaling, and maintenance of barrier integrity (Eltanani et al., 2022), a reduction in mitochondrial NDPK activity impairs the conversion of NDPs to NTPs, leading to an intracellular buildup of NDPs and disrupting normal endothelial function. This finding aligns with previous studies demonstrating that NDPK activity, encoded by the NME gene family, is diminished under hypoxic and hyperglycemic conditions (Qiu et al., 2016; Onyenwoke et al., 2012), limiting NTP production and leading to NDP buildup within the cell. The importance of NDPK in endothelial function is further underscored by studies showing that NDPK knockout mice exhibit compromised vascular integrity (Chatterjee et al., 2020).

The second mechanism that could explain the accumulation of NDPs in HRECs experiencing the Warburg effect induced by HG and Hyp is the increased activity of nucleoside triphosphate hydrolases (NTPases), enzymes that hydrolyze NTPs into NDPs. Both Hyp and HG are known to upregulate NTPase activity as a cellular adaptation to stress (Pimentel et al., 2013; Lunkes et al., 2008). This increased activity of NTPase accelerates the breakdown of NTPs into NDPs, further contributing to their accumulation.

In addition to impaired NDP-to-NTP conversion and enhanced NTP hydrolysis, our multi-omics analysis identified increased nucleotide biosynthesis as a significant contributor to NDP accumulation under HG and Hyp. The upregulation of several key biosynthetic enzymes suggests elevated production of nucleotide precursors, thereby expanding the intracellular nucleotide pool and amplifying NDP accumulation under these stress conditions. For instance, our metabolomic data showed elevated levels of IMP, a key precursor in the purine synthesis pathway, under HG+Hyp (Figure 2B), consistent with previous reports in diabetes (Luo et al., 2022) and Hyp (Sahlin and Katz, 1989). Furthermore, our proteomic data indicated that IMP accumulation may arise from multiple sources. One important route involves GMP conversion via GMP reductase (GMPR), which was upregulated under HG+Hyp (Figure 6B). Additional contributions come from increased activity in the *de novo* synthesis and salvage pathways. Within the *de novo* purine biosynthesis pathway, several enzymes, including PFAS, ADSS2, and ATIC, were upregulated, suggesting enhanced flux through this pathway and potentially expanding both IMP and AMP pools. The purine salvage pathway was also activated, as evidenced by the upregulation of adenine APRT and GMPR, indicating increased recycling of adenine and GMP into AMP and IMP, respectively. In contrast, HPRT1 and purine nucleoside phosphorylase (PNP) remained unchanged. Interestingly, IMPDH1, the enzyme responsible for converting IMP to XMP and subsequently to GTP, was downregulated, potentially creating a metabolic bottleneck that favors GDP accumulation over GTP synthesis. These findings collectively indicate a rerouting of purine metabolism toward AMP production, contributing to the imbalance in nucleotide pools observed in HRECs under HG+Hyp and in vitreous samples from patients with PDR.

In support, we observed a shift in adenine nucleotide metabolism in HRECs under HG+Hyp, characterized by altered expression of adenylate kinases (Aks) and enzymes involved in AMP catabolism (Figure 7A). Specifically, AK2, the mitochondrial adenylate kinase responsible for catalyzing the reaction 2ADP ↔ AMP + ATP within the mitochondrial intermembrane space, was downregulated, suggesting impaired mitochondrial ATP regeneration. Interestingly, we also detected upregulation of AK4, a mitochondrial protein structurally related to adenylate kinases but lacking catalytic activity. While AK4 does not catalyze nucleotide reactions, it is thought to function as a nucleotide sensor or stress-response modulator, playing a role in cellular adaptation to metabolic stress. In contrast, AK1, the cytosolic isoform that catalyzes the same reaction as AK2, was upregulated. Under elevated AMP conditions, AK1 likely drives the backward reaction (AMP + ATP → 2 ADP), contributing to increased cytosolic ADP levels. Importantly, the high intracellular AMP levels can be attributed to multiple converging mechanisms. First, our proteomic analysis (Figure 6C) revealed a significant upregulation of MAT2A, which promotes adenosine production from methionine, providing a potential substrate for AMP synthesis. Second, we observed significant downregulation of AMPD2, the enzyme responsible for converting AMP to IMP, indicating impaired AMP catabolism. Additionally, as described earlier, both the *de novo* and salvage pathways for AMP biosynthesis were upregulated. These combined metabolic alterations result in elevated intracellular AMP levels to facilitate downstream AK1 activation and ADP buildup.

The combined accumulation of AMP and ADP may also activate AMP-activated protein kinase (AMPK), a central energy sensor known to promote glycolytic reprogramming under metabolic stress. Although our study did not directly assess AMPK activity, previous reports suggest that AMPK activation under high AMP+ADP to ATP ratios can inhibit NDPK activity (Onyenwoke et al., 2012). Such inhibition would further limit NTP production, exacerbating NDP accumulation. Thus, AMPK activation may represent an additional mechanism sustaining the Warburg effect and nucleotide imbalance in HRECs exposed to HG+Hyp.

In the pyrimidine pathway, we observed a shift from *de novo* synthesis to salvage metabolism. The *de novo* enzyme DHODH was downregulated, whereas salvage enzymes such as UCK2 and CMPK1 were upregulated, consistent with the observed accumulation of CDP and UDP under the Warburg effect induced by HG+Hyp.

It is worth mentioning that the accumulation of NDPs in HRECs under HG+Hyp is crucial for generating the deoxynucleotides necessary for nucleic acid synthesis during cellular proliferation and angiogenesis. Ribonucleotide reductase (RNR) is the only enzyme responsible for converting NDPs into deoxynucleoside diphosphates (dNDPs), which are then transformed into deoxynucleoside triphosphates (dNTPs) essential for DNA replication. The regulation of RNR activity is, therefore, critical to ensure the stable production of all four dNTPs required for DNA replication (Torrents, 2014). However, hypoxic conditions present a challenge to RNR function, as oxygen is an essential cofactor for the mammalian RNR subunits RRM1/RRM2. Interestingly, research by Foskolou et al. has shown that RNR retains its activity under Hyp by switching from the RRM2 subunit to the RRM2B subunit, which is more adept at functioning in low-oxygen environments (Foskolou et al., 2017). This switch allows endothelial proliferation, particularly in the context of the Warburg effect induced by HG and Hyp, where cells rely heavily on glycolysis and are frequently exposed to hypoxic conditions. Our proteomic data (Figure 7) support this observation by showing that RRM2 and RRM2B are both increased during the Warburg effect induced by HG+Hyp. Additionally, maintaining an elevated ADP/ATP ratio in the cytoplasm has been suggested to inhibit mitochondrial function and further promote glycolysis (Maldonado and Lemasters, 2014). This shift creates a feedback loop, or a “vicious cycle,” where glycolysis continuously supplies substrates necessary for RNR activity.

**FIGURE 9:**
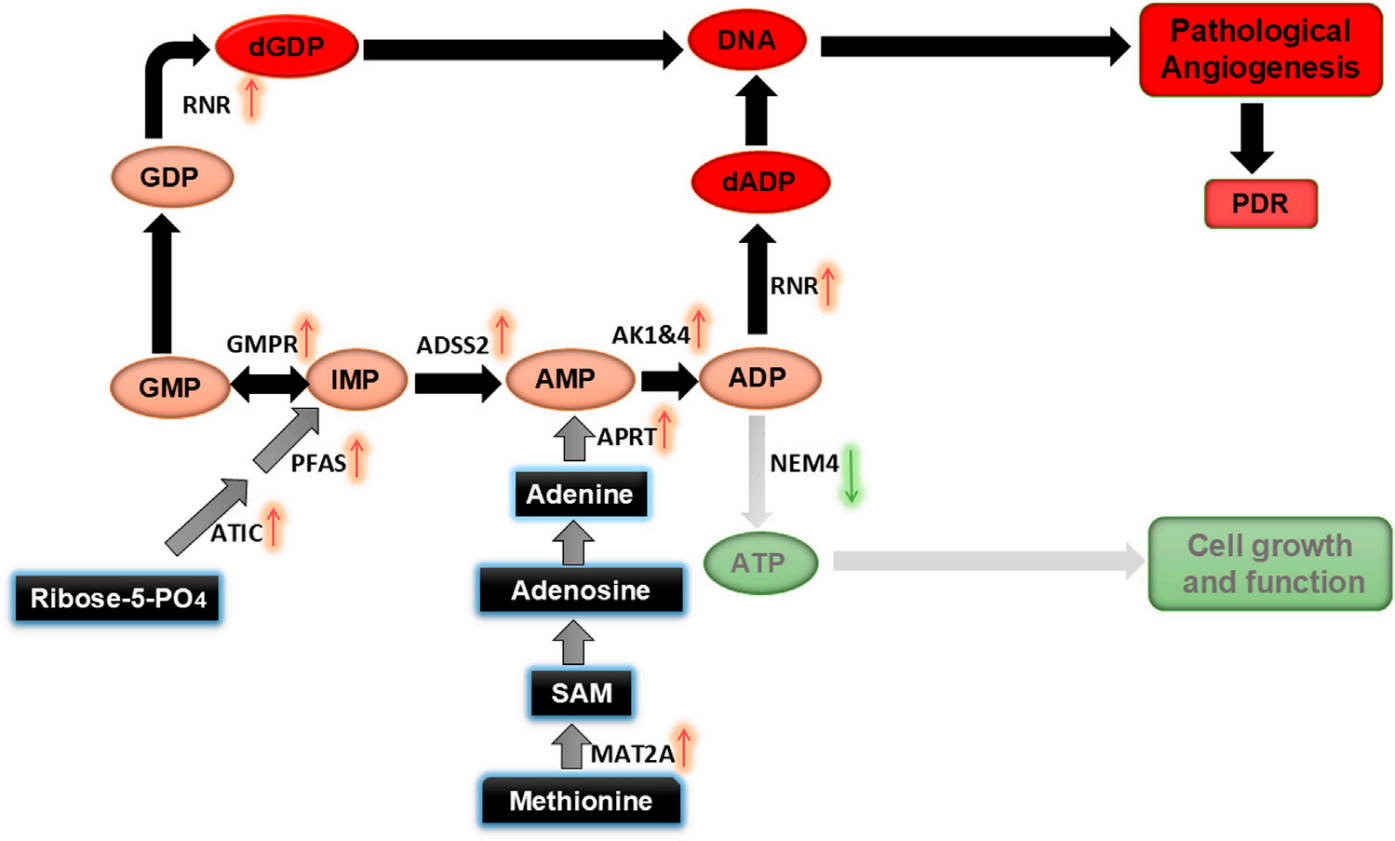
Proposed mechanism for altered nucleotide metabolism induced by HG and Hyp, leading to pathological retinal angiogenesis in PDR.

In summary, the combined effect of HG and Hyp triggers distinct metabolic reprogramming in HRECs, leading to NDP accumulation and pathological angiogenesis, a hallmark of PDR. Our multi-omic analysis reveals that reduced mitochondrial NDPK activity, increased cytosolic AK, and enhanced nucleotide precursor production disrupt nucleotide balance, resulting in NDP buildup and a scarcity of NTPs. This imbalance creates energy deficits in HRECs, impairing the normal endothelial function of maintaining blood vessel barrier integrity and driving a compensatory shift toward angiogenesis to restore oxygen and nutrient supply to stressed retinal tissues (Figure 9). These findings align with the bioenergetic differences between angiogenic and non-angiogenic endothelial cells, wherein the Warburg effect in proliferating angiogenic cells provides carbon backbones necessary for biomass formation and reductive biosynthesis, whereas oxidative phosphorylation in normal cells fully converts glucose and other substrates to CO_2_ and H_2_O (Vander Heiden et al., 2009). Importantly, the accumulation of NDPs supports their conversion into dNTPs required for nucleic acid synthesis during angiogenesis.

In conclusion, our study sheds light on the metabolic disturbances in HRECs under HG and hypoxic conditions, which contribute to retinal endothelial angiogenesis in PDR. The observed accumulation of NDPs, coupled with an elevated AMP+ADP pool in the vitreous humor of PDR patients, provides compelling evidence of disturbed nucleotide metabolism in PDR pathogenesis. These findings highlight the potential of monitoring the AMP+ADP pool as a biomarker for early PDR detection and suggest targeting nucleotide metabolism pathways as a therapeutic strategy to mitigate pathological angiogenesis in PDR.

### Limitations and future directions

Although this study integrates LC-MS/MS-based metabolomic profiling, proteomic analysis, *in vitro* angiogenesis assays, and human vitreous sample validation to provide novel insights into nucleotide dysregulation in PDR, several limitations should be acknowledged. First, the biological sample size for *in vitro* HRECs (n = 4 per group) may limit statistical power for some comparisons; however, the consistent trends across metabolomic and proteomic datasets support the overall coherence of our findings. Increasing the number of biological replicates in future studies will further strengthen statistical confidence. Second, while glucose was the primary carbon source examined under HG and Hyp, other substrates such as glutamine are also known to contribute to nucleotide biosynthesis, particularly under hypoxic conditions. Future experiments using isotope-labeled metabolic flux analysis will be important to delineate the relative contributions of glucose, glutamine, and other anaplerotic inputs to nucleotide synthesis. Third, although proteomic data support alterations in nucleotide metabolic pathways, protein abundance does not always reflect enzymatic activity, which is frequently regulated post-translationally. Follow-up enzyme activity assays and genetic or pharmacological inhibition of the identified enzymes will be necessary to validate their mechanistic roles in driving angiogenesis. Fourth, vitreous samples contain heterogeneous cellular and extracellular components, which may confound interpretation; nonetheless, our parallel *in vitro* HREC experiments strengthen the link between the metabolic alterations observed in vitreous and endothelial-driven pathological angiogenesis. Additional studies using cell-type–specific approaches (e.g., single-cell multi-omics) will help clarify the contributions of different vitreous cell populations to the observed changes. Finally, while ROC analysis identified vitreal AMP+ADP as a potential discriminator of PDR, the proposed cutoff value (0.0062 μM) should be interpreted cautiously, given the limited cohort size. Larger, independent validation studies will be necessary to establish reproducibility and to evaluate whether AMP+ADP levels are informative in more clinically accessible biofluids such as plasma or serum.

## Data Availability

The original contributions presented in the study are publicly available. This data can be found here: https://doi.org/10.6084/m9.figshare.30341782.
