# Correction: Impact of the Warburg effect on nucleotide homeostasis in human retinal endothelial cells and its relevance to proliferative diabetic retinopathy

**DOI:** 10.3389/fphar.2026.1907892

**Published:** 2026-07-14

**Authors:** Andrew Gregory, Ahmed M. Awad, Shaimaa Eltanani, Thangal Yumnamcha, Jenna Hart, Rao Me, Xihui Lin, Mohamed Shawky, Ahmed S. Ibrahim

**Affiliations:** 1 Department of Ophthalmology, Visual, and Anatomical Sciences, Wayne State University, Detroit, MI, United States; 2 Department of Pharmacology and Toxicology, Faculty of Pharmacy, Mansoura University, Mansoura, Egypt; 3 Department of Pharmacology and Toxicology, Faculty of Pharmacy, Mansoura National University, Mansoura, Egypt; 4 Department of Pharmacology, School of Medicine, Wayne State University, Detroit, MI, United States; 5 Molecular Therapeutics Research Program, Karmanos Cancer Institute, School of Medicine, Wayne State University, Detroit, MI, United States

**Keywords:** proliferative diabetic retinopathy (PDR), the Warburg effect, nucleotide metabolism, human retinal endothelial cells (HRECs), high glucose (HG), hypoxia (Hyp), biomarkers, nucleoside monophosphates (NMPs)

Author Ahmed M. Awad was erroneously assigned to affiliation 1. This affiliation has now been removed for author Ahmed M. Awad.

Author Mohamed Shawky was erroneously assigned to affiliation Department of Biochemistry, Faculty of Pharmacy, Horus University, New Damietta, Egypt. This affiliation has now been removed for author Mohamed Shawky.

Author Ahmed S. Ibrahim was erroneously assigned to affiliation Department of Biochemistry, Faculty of Pharmacy, Mansoura University, Mansoura, Egypt. This affiliation has now been removed for author Ahmed S. Ibrahim.

Affiliation Department of Pharmacology, School of Medicine, Wayne State University, MI, United States was erroneously given as Department of Pharmacology, Wayne State University, Detroit, MI, United States.

The original article has been updated.

