# Supplementary material for: Impact of the Warburg effect on nucleotide homeostasis in human retinal endothelial cells and its relevance to proliferative diabetic retinopathy
Source: Front Pharmacol. 2025 Nov 3;16:1660067. doi: 10.3389/fphar.2025.1660067 (PMC12620409; doi:10.3389/fphar.2025.1660067)

Supplementary Figure 1: Pathway enrichment analysis of significantly altered metabolites in Human Retinal Endothelial Cells (HRECs) exposed to combined HG and Hyp.

Supplementary Figure 2: Experimental Workflow for HREC Culture and Treatment under HG and Hyp Conditions.

Supplementary Figure 3: Enhanced angiogenesis under the Warburg effect induced by HG and Hyp in HRECs. **(A)** Proteomic analysis of glucose transporter 1 (Glut1) and lactate dehydrogenase (LDH) expression. **(B)** Representative phase-contrast images showing tube formation on Matrigel. **(C)** Quantification of tube length. Data are presented as mean ± SD (n = 4 per group). Statistical comparisons were made between control and treatment groups: *p < 0.05, **p < 0.01, ***p < 0.001; ns: not significant.

Supplementary Table 1. Rate of proliferative diabetic retinopathy (PDR) in patients with vitreous AMP+ADP levels above or below the cutoff of 0.0062 μM (calculated by the ROC curves) compared with non-PDR controls.

ADP+AMP level	PDR		Non-PDR		OR[a]
	n	%	n	%	
**High**	3	75[b]	1	12.5	21
**Low**	1	25	7	87.5	

a: OR: Odds ratio. The likelihood of elevated ADP+AMP levels occurring in the PDR group compared to the control group, after excluding outliers identified by Grubbs' test.
b: p< 0.027 compared to the non-PDR group.

Supplementary Fig 1

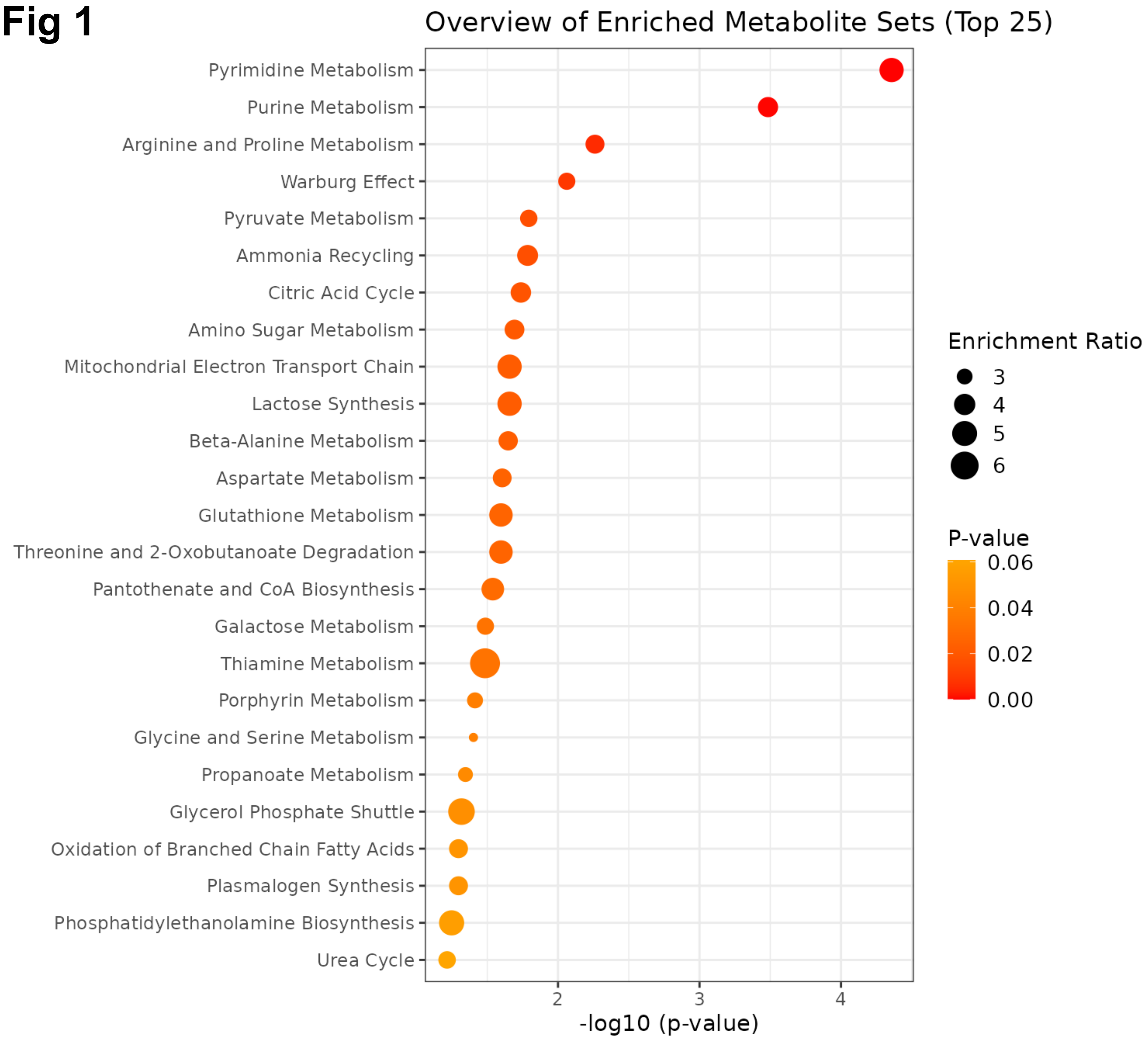

Supplementary Fig 2

- Step 1: Cell Source & Medium
  - HRECs from Cell Systems (Kirkland, WA, USA)
  - EGM-2 MV medium [5 mM D-glucose + 5% FBS + growth factors]

↓

- Step 2: Initial Culture
  - Passages 3–9; Seed in 100 mm Petri dishes, Grow to ~90% confluency

↓

- Step 3: Experimental Medium
  - Fresh medium with 5% FBS, no growth factors
  - Control: +25 mM mannitol (5 mM glucose)
  - High Glucose: +25 mM D-glucose (30 mM total)

↓

- Step 4: Treatment Conditions
  - Maintain 4 days under medium conditions, then 24 h exposure to:
  - Normoxia (21% O_2, 5% CO_2)
  - Hypoxia (2% O_2, 5% CO_2)

↓

- Step 5: Downstream Applications
  - Collect cells for metabolomics
  - Proteomics
  - Tube formation assay after transferring into Matrigel

Supplementary Fig 3

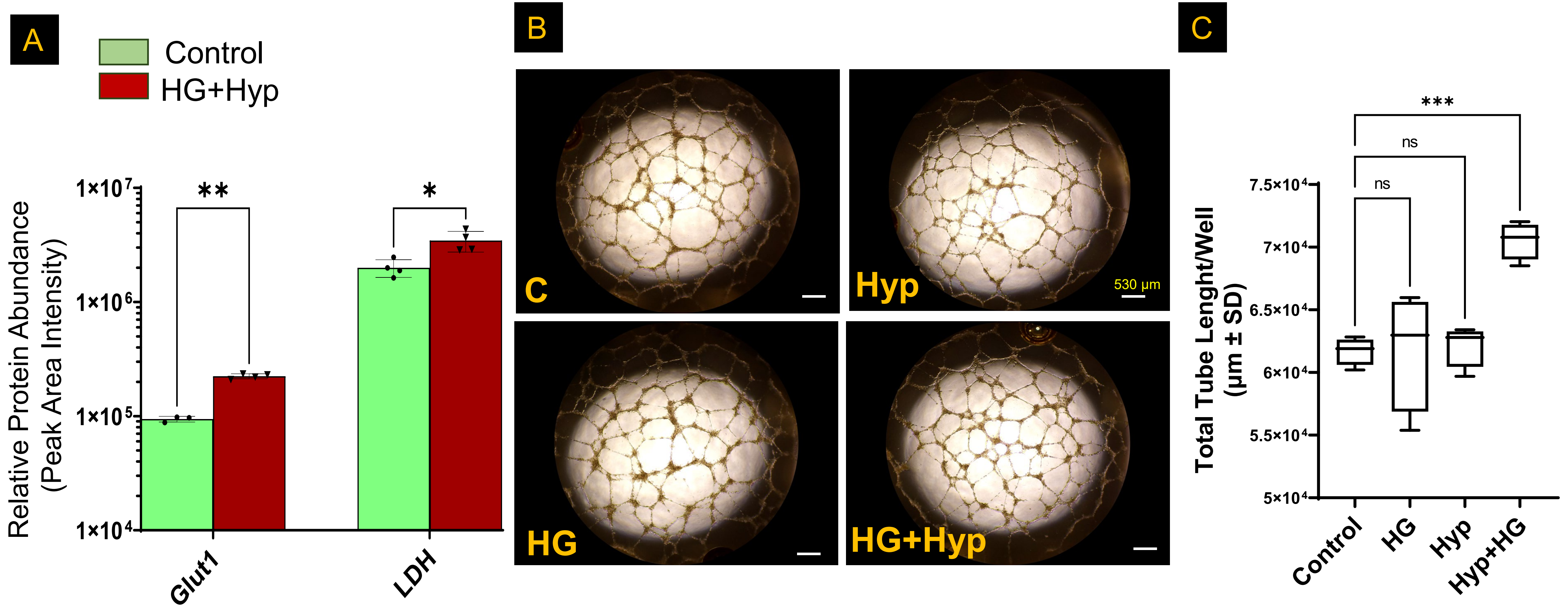

Supplement: Supplementary file 2 [file Presentation1.pdf]
